# Processing effects of L1/L2 from L3 in translation recognition paradigm: an exploratory ERP study

**DOI:** 10.3389/fpsyg.2025.1710959

**Published:** 2026-01-13

**Authors:** Karlygash Zhaksylykkyzy, Zhanar K. Ibrayeva, Almira M. Kustubayeva, Dietmar Roehm, Manzura Zholdassova

**Affiliations:** 1 Department of Turkology and Language Theory, Al-Farabi Kazakh National University, Almaty, Kazakhstan; 2 Brain Institute of Al-Farabi Kazakh National University, Almaty, Kazakhstan; 3 Department of Linguistics, Centre for Cognitive Neuroscience, University of Salzburg, Salzburg, Austria

**Keywords:** cross-linguistic influence, event-related potentials, N400, translation recognition paradigm, trilingualism

## Abstract

The complex dynamics of multilingual word recognition and the importance of considering language distance in the learning sequence are pivotal for comprehending multilingual language processing mechanisms. This study investigates brain activation during a translation recognition task, wherein trilingual individuals activate translation equivalents from English (L3) in both their first language (L1) and second language (L2) during EEG recording. Participants were categorized into two groups: (1) native speakers of Kazakh (L1K) with Russian as L2 and English as L3, (2) native speakers of Russian (L1R) with Kazakh as L2 and English as L3. Behavioral results revealed differentiation only in the accuracy data. ERP findings demonstrate that upon word presentation, participants distinguish between congruent and incongruent words, particularly evidenced by the modulation of the N400 component. Notably, the N400 amplitude effect was observed in the L1K and L1R groups for recognizing incongruent L1 and L2 words. These findings suggest that during L3 processing, trilinguals have access to previously acquired linguistic knowledge, albeit the interactivity level in the multilingual language system varies. Given the modest and imbalanced sample size, the study should be regarded as exploratory, providing the first ERP evidence from Kazakh-Russian-English trilinguals.

## Introduction

Understanding how multiple languages are represented and processed in the brain is a central question in psycholinguistics and cognitive neuroscience. While a substantial body of research has examined bilingual language processing, considerably less is known about the neural mechanisms underlying trilingual language use, particularly in sequential multilinguals. Studying trilinguals provides a unique opportunity to test and refine existing models of bilingual lexical organization by examining how previously acquired languages interact during the processing of an additional language. In this context, investigating how first (L1) and second (L2) languages are co-activated during third language (L3) processing can offer critical insights into cross-linguistic influence, language dominance, and the organization of the multilingual lexicon. However, neurophysiological evidence addressing these issues remains limited, especially for typologically distant language combinations.

The primary objective of the present study is to investigate how trilinguals process L1/L2 translations, which entail actively either L1 or L2 items that are semantically related to a word from their subsequently acquired L3. This would provide insight into whether a subsequently learned additional language influences the processing of the first and second language, and to what extent. Specifically, the research aims to determine the degree to which the acquisition and processing of the L3 influences the native language (L1) or the second language (L2) in the backward translation recognition task. The study focuses on individuals with Kazakh and Russian as their respective L1s, which are divided into two separate groups based on the language of schooling (mirror-image methodology) in the pre-experimental interview, while English serves as the targeted L3 language. Some research indicates that in the early stages of second language acquisition, learners primarily access the meaning of second language words through their first language. It is only with increasing proficiency that they develop the ability to directly engage in conceptual processing of the second language (Chen and Leung, 1989; Kroll and Curley, 1988). Furthermore, as fluency in the second language improves, this shift may lead to an imbalance in the strength of the connections between the lexicon and conceptual knowledge between the first and second languages. This asymmetry in the lexical-to-conceptual connections is discussed in many other translation studies (Kroll, 1993; Kroll and Sholl, 1992; Kroll and Stewart, 1994; Sholl et al., 1995; Keatley et al., 1992; Roufca, 1992; Sánchez-Casas et al., 1992).

In order to account for how bilingual individuals process lexical and conceptual information in their two languages, Kroll and Stewart (1990, 1994) introduced the Revised Hierarchical Model (RHM), which captures the aforementioned asymmetries. This model posits that both lexical and conceptual connections are active within bilingual memory. Still, the strength of these connections varies based on the individual’s fluency in the second language (L2) and the relative dominance of the first language (L1) compared to the second language. Furthermore, when an individual learns a second language in early childhood, a robust connection exists between the lexicon of their first language and their conceptual memory. In the initial phases of second language acquisition, second language words are primarily associated with this system through lexical connections to the first language. As proficiency in the second language improves, direct conceptual connections are also developed. However, the lexical connections do not diminish with the establishment of conceptual links, they continue to exist as germinative connections (De Groot, 1992).

Although the Revised Hierarchical Model does not explicitly incorporate age of acquisition as an independent parameter, age of acquisition is indirectly reflected through its effects on language proficiency, dominance, and the strength of lexical-conceptual connections. In the present study, participants acquired their third language (English) in a relatively similar educational context and age range, which limits variability in age of acquisition. Accordingly, age of acquisition was not treated as a primary factor in the analyses but is acknowledged as a relevant background variable that may modulate cross-linguistic interactions.

The RHM explains lexical organization in individuals who acquire languages sequentially while also acknowledging the fluid nature of bilingual proficiency. The model suggests that shifts in language dominance can lead to asymmetrical processing that favors the second language (Kroll et al., 2010). The RHM continues to be recognized as a resilient framework that can adapt to incorporate novel research discoveries, and in respect to this, an important aspect is to investigate the relationship between L1 and L2 in the interaction with the L3.

The recent research on the acquisition of a third language (L3) focuses on two main perspectives regarding the transfer of linguistic features, specifically examining which languages, L1 or L2, have a greater influence on L3 acquisition. One of these perspectives posits that the transfer of features occurs equally from both L1 and L2 to L3. This view finds support in a study conducted by Flynn et al. (2004), who investigated the transfer of features from Kazakh (L1) and Russian (L2) to English (L3). Their findings indicated that both L1 and L2 had an equal impact on the oral production of relative clauses in L3. This suggests that prior knowledge of both L1 and L2 is accessible during the acquisition of L3, indicating an equal influence of both languages on L3 acquisition.

The other perspective, known as the L2 Status Factor (Bardel and Falk, 2007; Bardel and Falk, 2012), argues that the transfer of features predominantly occurs from L2 to L3. Supporting this view, a study by Jaensch (2011) revealed that the transfer of features into German (L3) was dependent on proficiency in English (L2). These findings provide evidence for the L2 Status Factor, suggesting that L2 plays a more significant role in the transfer to L3 compared to L1. This perspective suggests that L2 acts as a ‘filter’, potentially making L1 less accessible. This can be attributed to the similarities between L2 and L3, as they are both learned languages rather than acquired languages like L1, as proposed by Paradis (1994, 2004, 2009). According to this paradigm, procedural memory is used to support implicit L1 learning of vocabulary and grammar. However, for L2, declarative memory is intended to be used in the explicit learning of vocabulary and grammar. It is anticipated that L2 learners would only become dependent on procedural memory in extreme circumstances and under implicit, communicative learning environments (Paradis, 1994, 2009).

Importantly, the present study focuses on lexico-semantic processing rather than morphosyntactic transfer. The influence of L1 and L2 on L3 is examined here in terms of semantic access and cross-language activation during translation recognition, as indexed by behavioral performance and N400 modulations. While other linguistic domains, such as morphology or syntax, may also shape L3 acquisition, these aspects were not directly tested in the current design and therefore fall outside the scope of the present investigation.

Language switching effects in trilinguals were effectively shown in Aparicio and Lavaur (2014) using non-cognate items in a traditional generalized lexical decision task. To better understand language impacts and cross-language semantic linkages, it is therefore interesting to examine language switching effects in trilinguals using a translation priming paradigm. Aparicio and Lavaur (2016) conducted a comprehensive investigation into masked translation priming effects in visual word recognition by trilingual individuals. The study aimed to elucidate how trilinguals process their non-dominant languages and the extent to which these languages influence each other, with a specific focus on the role of the dominant language in word recognition. The researchers recruited 24 French-English-Spanish trilinguals who were unbalanced in their language proficiency, yet deemed equivalent in their second (L2) and third (L3) languages. Participants performed lexical decision tasks in their non-native languages (L2 and L3) using a masked translation priming paradigm. Target words were primed by either the same word (repetition), a translation from one of the other languages, or an unrelated word in any of the three languages. The results of the study revealed a significant priming effect, with faster identification of target words when primed by the same word (repetition) in both L2 and L3. Importantly, a translation priming effect was observed only when the primes belonged to the dominant first language (L1). Trilinguals showed faster word recognition in L2 and L3 when primed by the L1 translation, indicating a strong influence of the dominant language on cross-language interactions. No translation priming effects were found for L2 and L3 primes, suggesting a lack of direct interactions between the non-dominant languages. Overall, the findings support the notion of a multilingual lexicon organized around the dominant language, with the dominant language playing a crucial role in facilitating word processing across languages.

Verb semantics processing in translation tasks involves understanding and managing the meaning of verbs during the translation process. This process is essential for accurately conveying the intended message from the source language to the target language. Balaguer et al. (2005) conducted an ERP study on regular and irregular verb processing in early bilinguals. The study aimed to investigate the influence of language similarity on the acquisition of morphosyntactic information in highly proficient Catalan-Spanish bilinguals. Sixteen highly proficient bilinguals were involved who were native speakers of Catalan and had learned Spanish as a second language at an early age. The participants were presented with Spanish verbs in three conditions: regular, semi-regular, and irregular. The researchers recorded ERPs while the participants performed a lexical decision task on the verbs. The results showed that the processing of regular and irregular verbs elicited different ERP patterns. Regular verbs elicited a larger P600 component, which is associated with syntactic processing, while irregular verbs elicited a larger N400 component, which is associated with semantic processing. The researchers suggest that this difference in ERP patterns reflects the different processing strategies used for regular and irregular verbs. Furthermore, the study found that the processing of irregular verbs was influenced by the degree of similarity between the two languages. Specifically, the bilinguals showed a larger N400 effect for irregular verbs that were similar in form and meaning across the two languages. This suggests that the processing of irregular verbs is influenced by the degree of overlap between the two languages.

An established technique for investigating how bilingual individuals comprehend the meaning of words in their language is the translation recognition task (De Groot, 1992). This task requires participants to assess whether a given second word in a different language is the accurate translation of a preceding word in the first language. In executing this task, participants must access the meaning of the initial word and subsequently compare it with the meaning of the second word.

In our current investigation, we adopted an existing translation priming task, previously employed to gauge participants’ discernment of both meaning and formal similarity in relation to correct translations of L3 words. The present study used event-related potentials (ERPs), Reaction Time (RT) and behavioral judgments (accuracy) of the participants with respect to a translation recognition task. Processing the translation equivalents of L3 words assists in identifying the role of the first and second languages. Further aims at assessing the organization of language and cognitive functions in the human brain. Research on trilinguals remains limited, particularly concerning ERP evidence on semantic connections across all three languages. However, bilinguals have an abundance of evidence pointing to the fact that semantic representations of translation equivalents are shared within the mental lexicon (Francis, 2005; Kroll and Tokowicz, 2005; Laxén and Lavaur, 2010; Aparicio and Lavaur, 2016). In addition, this experimental design allows us to examine different combinations of language switching. This study contributes valuable insights into the complex dynamics of multilingual word recognition and highlights the importance of considering language dominance in understanding multilingual language processing mechanisms.

### Language information

The language situation in Kazakhstan is unique for various reasons; the formation of the language situation was influenced by many factors that are described in details in the sociolinguistic works of scientists (Suleimenova, 2011, 2020; Smagulova, 2021; Fierman, 2005, 2006; Alisharieva et al., 2017; etc.) The uniqueness of the language situation is in the long-term coexistence of two languages – Kazakh and Russian, which are representatives of different language families and have different structural and typological features. The Kazakh language belongs to the Turkic group (Kypchak-Nogai subgroup) of the Altaic language families. The Russian language is a representative of the East Slavic branch of the Slavic group of the Indo-European language families. The structural and typological characteristics of the analyzed languages are important factors in explaining many aspects of Kazakh-Russian and Russian-Kazakh bilingualism. The sequential attachment of one or more affixes to the root of a word is one of the leading indicators of the Kazakh language as an agglutinative type.

Kazakhs have the highest rates of multilingualism and proficiency in Russian. The overwhelming majority of Kazakhs are bilinguals and multilinguals, some of whom, due to their immersion in the remaining Russian communicative space and due to the inertia of their communicative habits, continue to communicate in Russian (Suleimenova, 2009). In modern Kazakhstan, there is a linguistic shift toward the Kazakh language. The reform of the linguistic education system (support for the Kazakh language, introduction of programs in English) influenced the language situation. However, the Russian language, the legal status of which is enshrined in the Constitution of the Republic of Kazakhstan, is a compulsory subject in schools and in the higher education system, where instruction is conducted in Kazakh and Russian, depending on the choice of language of instruction.

The present research is designed as an exploratory investigation into the dynamics of multilingual lexical processing using event-related potentials. While the sample size is modest relative to current recommendations for confirmatory EEG research, this study provides the first ERP evidence from Kazakh-Russian-English trilinguals, a population rarely represented in the neurolinguistic literature. The exploratory nature of this work allows us to identify patterns of neural activation and behavioral performance that can inform both theoretical models of multilingual processing and the design of future large-scale studies.

Based on these linguistic profiles and the theoretical frameworks reviewed above, we formulated the following hypotheses and questions:

*RQ1*: Are there any differences when processing L3 translations with respect to L1s and L2s? Is there an effect of the language learning sequence of L3?

*H1*: For L1R, the effect should be more pronounced for L2 relative to L1 because of *the distance in the language learning sequence*. Meanwhile, in the L1K group we expect equal effect for L2 relative to L1.

*RQ2*: Does the processing of L3 translations stimulate the same ROIs in both groups?

*H2*: Considering the H1 (the *distance in the language learning sequence*), we expect the asymmetry in ROIs within and between the L1K and L1R groups.

## Materials and methods

### Participants

Thirty-eight right-handed students were recruited through the advertisements at Al-Farabi Kazakh National University (Almaty, Kazakhstan) where they were studying at the time of the testing. Kazakh native speakers (L1K) [*n* = 22, six males, mean age = 23.95 (sd = 3.9) years, age range 19–32] and the Russian native speakers (L1R) [*n* = 16, four males, mean age = 24.44 (sd = 2.6) years, age range 18–29] having acquired English as a third foreign language (chronologically L3), subsequently after Kazakh/Russian as L2. Participants were interviewed before the main experiment to gather qualitative information on their linguistic backgrounds, including the sequence of language acquisition and daily language use. This process ensured that participants were grouped based on comparable linguistic profiles and educational exposure. While these interviews provided valuable insights, future studies will incorporate standardized language proficiency tests and comprehensive language background questionnaires to further strengthen group matching. All the subjects were Kazakh-Russian and Russian-Kazakh bilinguals with non-fluent English and had a higher command in their L2 than in the L3. At the time of the data elicitation, they were all students who had passed the official English language entrance exam that required them to have reached the B1/B2 level in English (as determined by the Common European Framework of References for Languages). All participants began learning English as a foreign language at a comparable age, starting from the third grade of primary school, as part of the national educational curriculum. In contrast, the second language (Kazakh or Russian) was acquired earlier through naturalistic and immersive exposure in the social and educational environment, resulting in a learning context more similar to first language acquisition. Information on the age of acquisition was obtained through pre-experimental interviews focusing on participants’ educational histories. At the time of testing, participants used both Kazakh and Russian in their daily lives and were relatively fluent in their second language, whereas English functioned as a non-fluent third language. This linguistic profile is characteristic of sequential trilinguals and facilitates comparisons between Kazakh-Russian-English and Russian-Kazakh-English language combinations. They participated voluntarily, and all signed an informed written research consent form prior to the experiments. The study was approved by the Local Ethics Committee of Al-Farabi Kazakh National University.

### Materials

Verbs were selected as target stimuli because they place high demands on semantic integration and conceptual processing, making them particularly suitable for investigating lexico-semantic access in translation recognition tasks. Previous psycholinguistic and ERP research has shown that verb processing reliably elicits N400 effects that are sensitive to semantic congruency (e.g., Balaguer et al., 2005). In the present study, verbs were treated as lexical-semantic units rather than as morphosyntactic paradigms. To minimize ambiguity, all verbs were presented in their infinitive form, and phrasal verbs as well as highly polysemous items were excluded from the stimulus set.

Given the cross-linguistic nature of the materials, it was also important to consider language-specific properties of the compared languages. While Kazakh and Russian share the Cyrillic writing system, Kazakh includes additional letters representing language-specific phonemes. These orthographic differences were taken into account in the selection and matching of the stimuli.

In the experiment, 105 irregular verbs (infinitives) of English were used. The English irregular verbs were categorized *a priori* into three groups based on their formal inflectional complexity, following standard pedagogical classifications. Group 1 included verbs with identical base, past tense, and past participle forms (e.g., cut-cut-cut). Group 2 comprised verbs with identical past tense and past participle forms but a distinct base form (e.g., build-built-built). Group 3 consisted of verbs with three distinct forms (e.g., sing-sang-sung). Although all verbs were presented in their infinitive form in the experiment, this categorization reflects differences in underlying formal and mnemonic complexity associated with the verb paradigm rather than semantic difficulty. This categorization reflects differences in formal and mnemonic complexity rather than semantic difficulty. This approach is consistent with psycholinguistic accounts linking inflectional irregularity and formal variability to increased memory demands and processing complexity (Bybee, 1995; Pinker and Ullman, 2002). 210 Kazakh words and 210 Russian words were used in the experiment. Half of them were Kazakh and Russian translation equivalents of the English irregular verbs and served as the congruent translation equivalents, while the other half were that served as incongruent translations for the control condition. All the incongruent translation words were matched to the congruent translation words in the mean length of 6 letters for Kazakh and 7 letters for Russian. A full list of stimuli with their characteristics is provided in the Appendix.

### Procedure

Participants in the L1 Kazakh group primarily received instruction through Kazakh (L2), while participants in the L1 Russian group received instruction through Russian (L1). Event-related potentials were recorded as Kazakh-Russian-English and Russian-Kazakh-English trilinguals decided whether any of the four candidate words was the correct translation of the English prime word by pressing a response button. Stimuli included both congruent translation equivalents and incongruent distractors. For example, the English verb *go* was paired with its correct translations (Kazakh *бару*, Russian *идти*) as well as semantically unrelated words. Incongruent distractors were manually selected to be conceptually unrelated to the English prime and its correct translations, and all distractor items were non-cognates across the three languages. This ensured that responses required active evaluation of meaning rather than form alone. The translation of words was presented randomly to eliminate the order effect. There was one experimental block consisting of 105 trials overall, where 105 congruent Kazakh and Russian translation equivalents and 105 incongruent Kazakh and Russian non-translation words. The ERPs generated by incongruent words that were non-cognates in all three languages were analyzed to examine processing differences across languages. The N400 is a negative deflection in the ERP peaking 400 ms after the onset of a stimulus sensitive to context or reduced processing of the stimulus.

**Figure 1:**
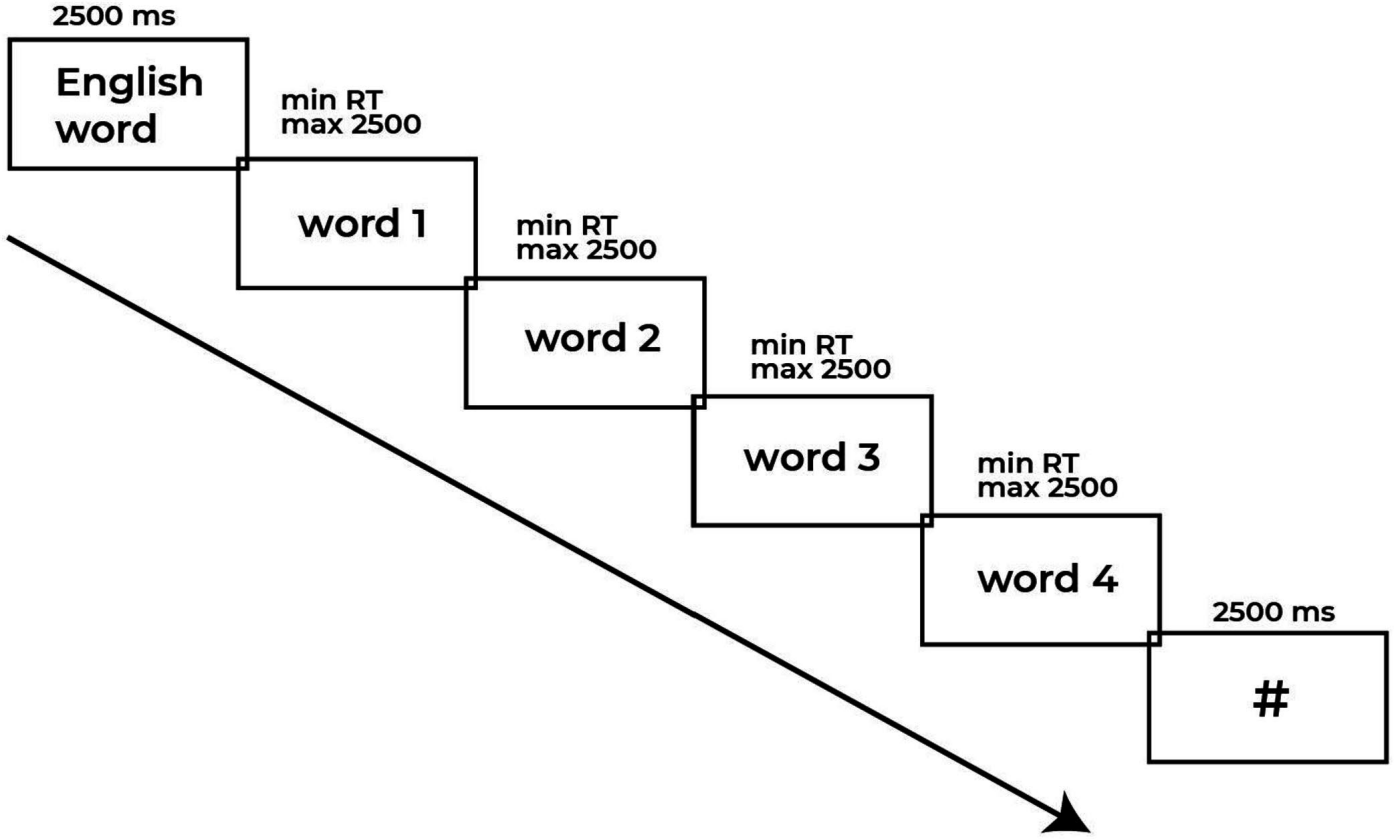
Example of a sequence of stimuli, here an English irregular verb (English word) followed by a random L1/L2 translation equivalents or incongruent words.

After the instructions were fully understood, the participants were presented with a training block of six trials. Following the training block, the participants performed the experimental block with the next sequence: irregular verb was presented in the center of the computer screen with black text on a white background for fixed 2,500 ms and replaced by either congruent or incongruent translations. Participants were instructed to fix their decision by pressing the key ‘1’ on a keyboard if the word was congruent translation equivalent and ‘3’ if the word was incongruent. Congruent and incongruent items were matched for frequency in Russian and Kazakh to control for lexical familiarity effects (Brysbaert and New, 2009). The time window for the button press was restricted to 2,500 ms. After each trial there was a fixation cross which appeared in the center of the screen for 2,500 ms until the next trial would be presented automatically. The presentation of four words following the L3 prompt aimed to minimize predictability and maintain participant engagement across trials. Reaction time (RT) was recorded from the onset of target presentation until the participant pressed the button (see Figure 1). The experiment was administered on a Windows 10 IntelR Core i-5 CPU computer with a 13-inch HD monitor (60 Hz refresh rate) using the E-Prime 2.0 software package (Psychology Software Tools Inc., 2012). Participants were seated in a quiet environment at approximately 60 cm from the computer screen.

### EEG recording and preprocessing

Participants were seated in a quiet room and performed the translation recognition task. Continuous EEG data were recorded during the task performance with a 64-channel (ANT-neuro, Germany). The layout for the 64 Ag/AgCl electrodes followed the standard International 10–20 montage system with intermediate locations. To monitor for blinks and saccades the electrode Fp1 was chosen to control for vertical eye movements (similar to typical VEOG placement) and another electrode AF7 was chosen to control for horizontal eye movements (similar to typical HEOG placement). Recordings were referenced online to AF8 but re-referenced offline to linked mastoids (M1 and M2). The mean of AFz and CPz electrodes served as the ground electrode. The default bandpass filter for raw data recording was set between 0.016 and 200 Hz, and the sampling rate was 1,000 Hz. The impedances of all electrodes were kept under 5 kΩ.

Brain Vision EEG data processing was performed using the computer software BrainVision Analyzer (version 2.0.1.). The preprocessing of the EEG data involved the filtering with a 0.1–30 Hz bandpass filter, afterwards, the EOG artifacts were corrected by Independent Component Analysis (ICA). The components identified as responsible for eye movement artifacts were removed from the raw data. Subsequently, each participant’s signal underwent visual scrutiny to further ensure accuracy and manual rejections were employed. Only trials devoid of muscle artifacts and eye movement/blink activity were selected for the subsequent averaging analysis. The data finally were epoched from – 200 to 1,000 ms relative to the onset of the critical second NP, baseline correction. Time windows were determined based on prior literature and pilot data, focusing on established N400 timeframes (400–600 ms; Kutas and Federmeier, 2011).

### Data analysis

We used R Version 4.1.2 (2021-11-01) for all statistical analyses (RStudio Team, 2021) and the packages tidyverse version 1.3.2 (Wickham et al., 2019), lme4 version 1.1–30 (Bates et al., 2015), car version 3.1–0 (Fox and Weisberg, 2011; Fox and Weisberg, 2019), emmeans version 1.8.1–1 (Lenth, 2019), and cowplot version 1.1.1 (Wilke, 2019). To produce model output tables, we used lmerOut version 0.5 (Alday, 2018) and kableExtra version 1.1.0 (Zhu, 2019). For all analyses below, contrasts for categorical factors used sum coding (for a tutorial on contrast coding, see Schad et al., 2020), i.e., coefficients reflect differences to the grand mean.

For clarity, the following abbreviations are used in the statistical models: group (L1 Kazakh vs. L1 Russian), cong (congruency: congruent vs. incongruent translations), lang (language of the translated word: Kazakh vs. Russian), pos (stimulus position within the trial), verb (verb group based on formal inflectional complexity: easy, medium, difficult), roi (region of interest), and chan (electrode channel).

### Behavioral data

The analysis was conducted using Generalized Linear Mixed Models (GLMM), which inherently evaluate fixed effects with fixed effects group, verb, position (pos), language (lang), congruency (cong) and their interactions while accounting for random variability across participants and items. This approach provides robust statistical testing of our hypotheses. The results focus on significant interactions and main effects derived from the model output. More complex random effect structures involving random slopes by participant and item did not converge.

### EEG data

Single-trial EEG data were analyzed using mixed effects models with fixed effects for group, verb, position (pos), language (lang), congruency (cong), ROIs, channels (chan) and their interaction for mean amplitude values per time window. Time windows were chosen on the basis of visual inspection of the data. Electrode channels were grouped into nine regions of interest (ROIs) by averaging signals across predefined clusters. This approach ensured no collinearity between channels and ROIs in the model. Each ROI, containing the averaged values from a group of five or six electrodes, was computed by collapsing data across the following electrodes: anterior left (AL, including ‘F7’, ‘F5’, ‘F3’, ‘FC7’, ‘FC5’, ‘FC3’), central left (CL, including ‘T7’, ‘C5’, ‘C3’, ‘TP7’, ‘CP5’, ‘CP3’), posterior left (PL, including ‘P7’, ‘P5’, ‘P3’, ‘PO7’, ‘PO5’, ‘PO3’, ‘O1’), anterior middle (A, M including ‘F1’, ‘Fz’, ‘F2’, ‘FC1’, ‘FCz’, ‘FC2’), central middle (CM, including ‘C1’, ‘Cz’, ‘C2’, ‘CP1’, ‘CP2’), posterior middle (PM, including ‘P1’, ‘Pz’, ‘P2’, ‘POz’, ‘Oz’), anterior right (AR, including ‘F4’, ‘F6’, ‘F8’, ‘FC4’, ‘FC6’, ‘FT8’), central right (CR, including ‘C4’, ‘C6’, ‘T8’, ‘CP4’, ‘CP6’, ‘TP8’), and posterior right (PR, including ‘P4’, ‘P6’, ‘P8’, ‘PO4’, ‘PO6’, ‘PO8’, ‘O2’). Models also included random slopes by participant and by item. More complex random effects structures including trials led to convergence problems.

## Results

### Behavioral results

**Figure 2:**
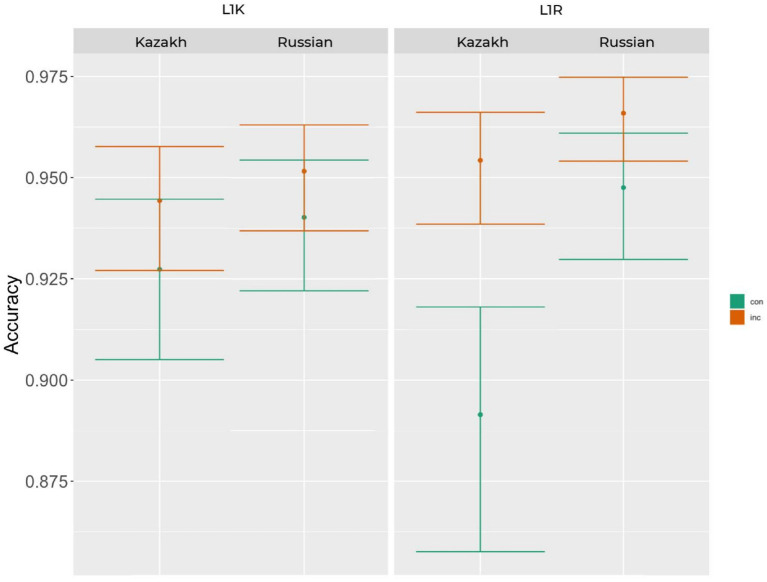
The mean accuracy rate of the interaction between congruency and group in the L1K and L1R groups including the language effect.

The mean accuracy rate for the L1 Kazakh group was 87% (sd = 34%) and for the L1 Russian group 87% (sd = 33%). Generalized linear mixed models were performed on the accuracy data. Iterative model fits revealed that the best fitting model for the data includes log-transformed RT as predictor and by-subject and by-item random intercepts as well as by-subject random slopes and by-item random slopes for congruency [glmer(acc ~ 1 + cong*log(rt + 1)*group + lang + pos + verb + (1 + cong|subj) + (1 + cong|item))]. The type II Wald test revealed main effects of cong [χ^2^ (1) = 16.29, *p* < 0.001], log(rt + 1) [χ^2^ (1) = 44.17, *p* < 0.001], lang [χ^2^ (1) = 62.66, *p* < 0.001], pos [χ^2^ (1) = 36.08, *p* < 0.001], verb [χ^2^ (1) = 20.47, *p* < 0.001], as well as an interaction between RT and cong [χ^2^ (1) = 28.71, *p* < 0.001], cong and group [χ^2^ (1) = 5.41, *p* < 0.05]. The result of the accuracy rate of the interaction between cong and group is visualized in Figure 2 using errorbar plots (Allen et al., 2019). The errorbars in this and the following figures represent 83% confidence intervals, the non-overlap of which corresponds to significance at the 5% level.

**Figure 3:**
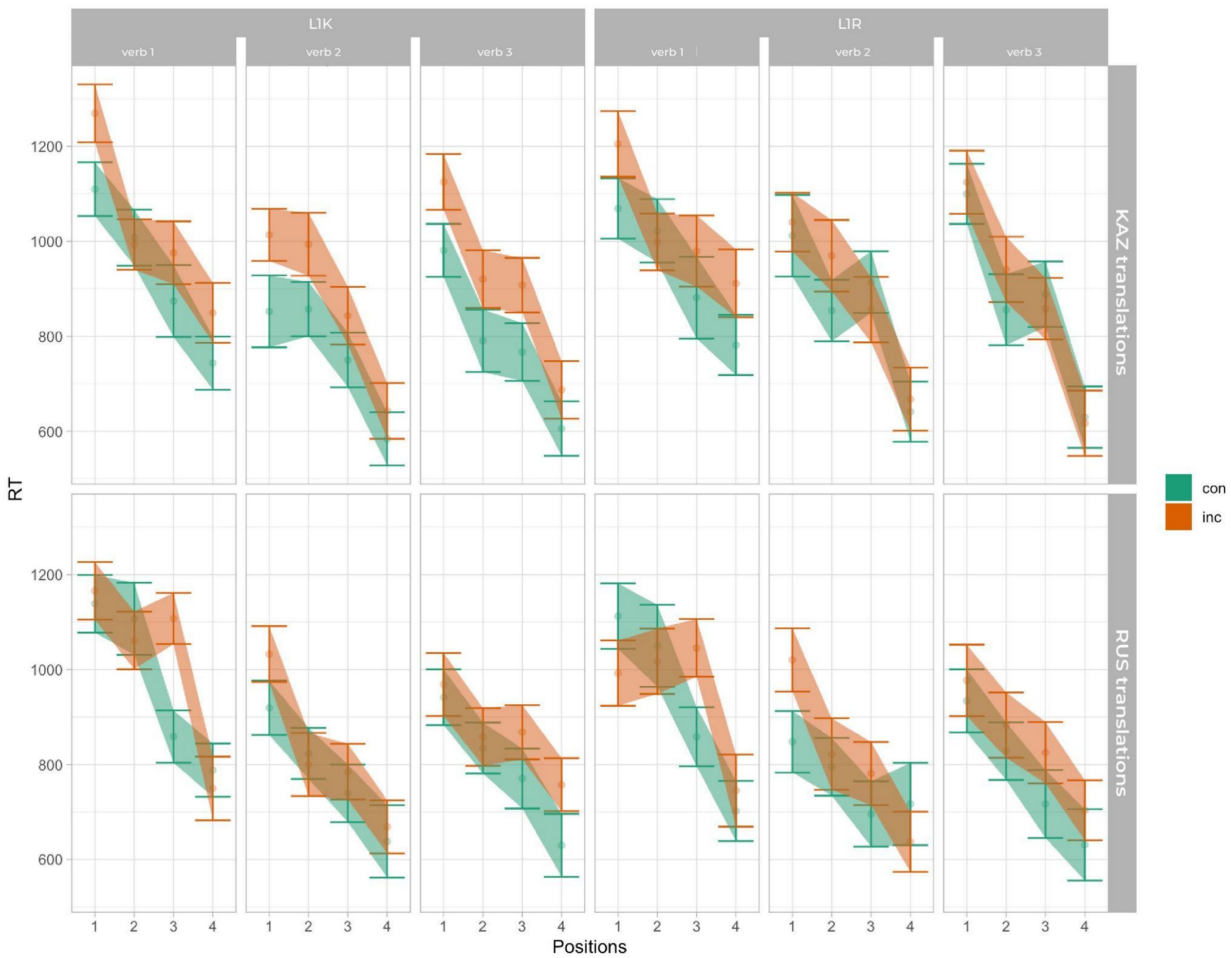
The mean reaction time.

The mean reaction time for the L1 Kazakh group revealed 879.63 (sd = 437.79) and for the L1 Russian group 876.81 (sd = 484.27). Linear mixed models were performed on reaction time data. The type II Wald test revealed the main effects of cong [χ^2^ (1) = 113.06, *p* < 0.001], lang [χ^2^ (1) = 16.74, *p* < 0.001], pos [χ^2^ (3) = 1510.70, *p* < 0.001], verb [χ^2^ (2) = 40.06, *p* < 0.001], as well as an interaction between cong:lang:verb:pos [χ^2^ (6) = 13.55, *p* < 0.05]. The result of the reaction time is visualized in Figure 3 (Allen et al., 2019). Figure 3 shows longer reaction times for incongruent compared to congruent trials across languages, with modulation by verb group and stimulus position, consistent with the significant main effects and higher-order interaction observed in the model. Reaction times also decreased across stimulus positions, indicating faster responses for later candidate words. Importantly, the size of the congruency difference varies across verb groups and positions: the separation between congruent and incongruent conditions is more pronounced at earlier positions and becomes smaller at later positions for some verb groups, consistent with the significant congruency × language × verb × position interaction observed in the model. The errorbars in this and the following figures represent 83% confidence intervals, the non-overlap of which corresponds to significance at the 5% level.

In respect of accuracy and RT when judging translations in both conditions in L3 English, we predicted that both groups would judge all translations faster (shorter RT) and obtain higher accuracy scores in their L1. Our results show that there is a complex interaction of different factors (as indicated by the significant interaction between RT, and congruency). The results cannot be a typical speed-accuracy trade-off effect otherwise, one would expect exactly the opposite pattern (the less accurate judgments in the incongruent translations, the faster RT). Therefore, this shows a processing difficulty that caused slower decisions and judgments that are more incorrect for both groups.

### ERP results (N400 amplitude)

**Figure 4:**
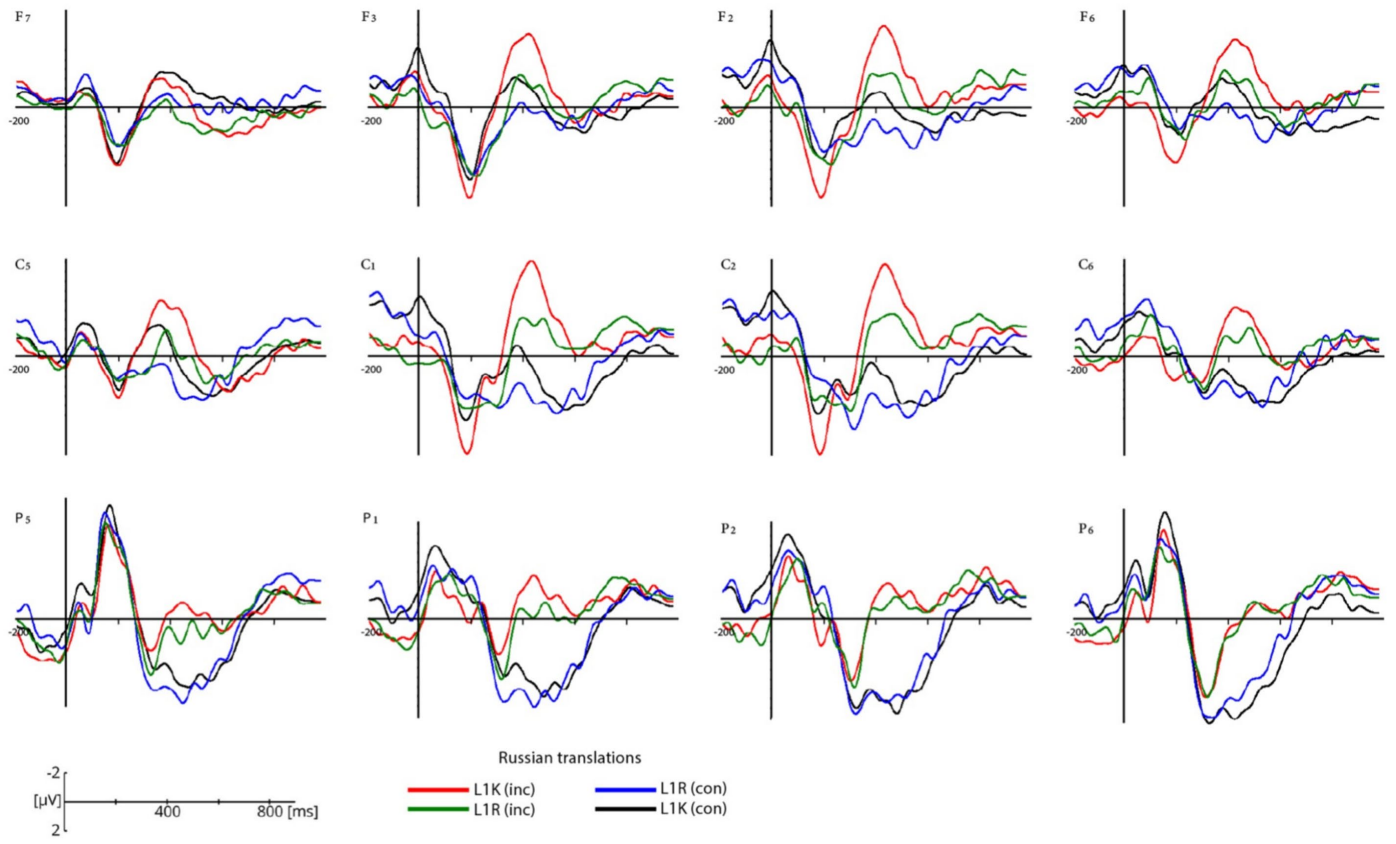
Grand average ERP waveforms for congruent Russian translations in the L1K group (black lines), congruent Russian translations in the L1R group (blue lines), and for incongruent Russian translations in the L1K group (red lines), for incongruent Russian translations in L1R group (green lines). ERPs are plotted for twelve electrodes (F7, F3, F2, F6, C5, C1, C2, C6, P5, P1, P2, P6) within each region of interest. The negative voltage is plotted up. Data are plotted from 200 ms prior to and 1,000 post stimulus onset.

**Figure 5:**
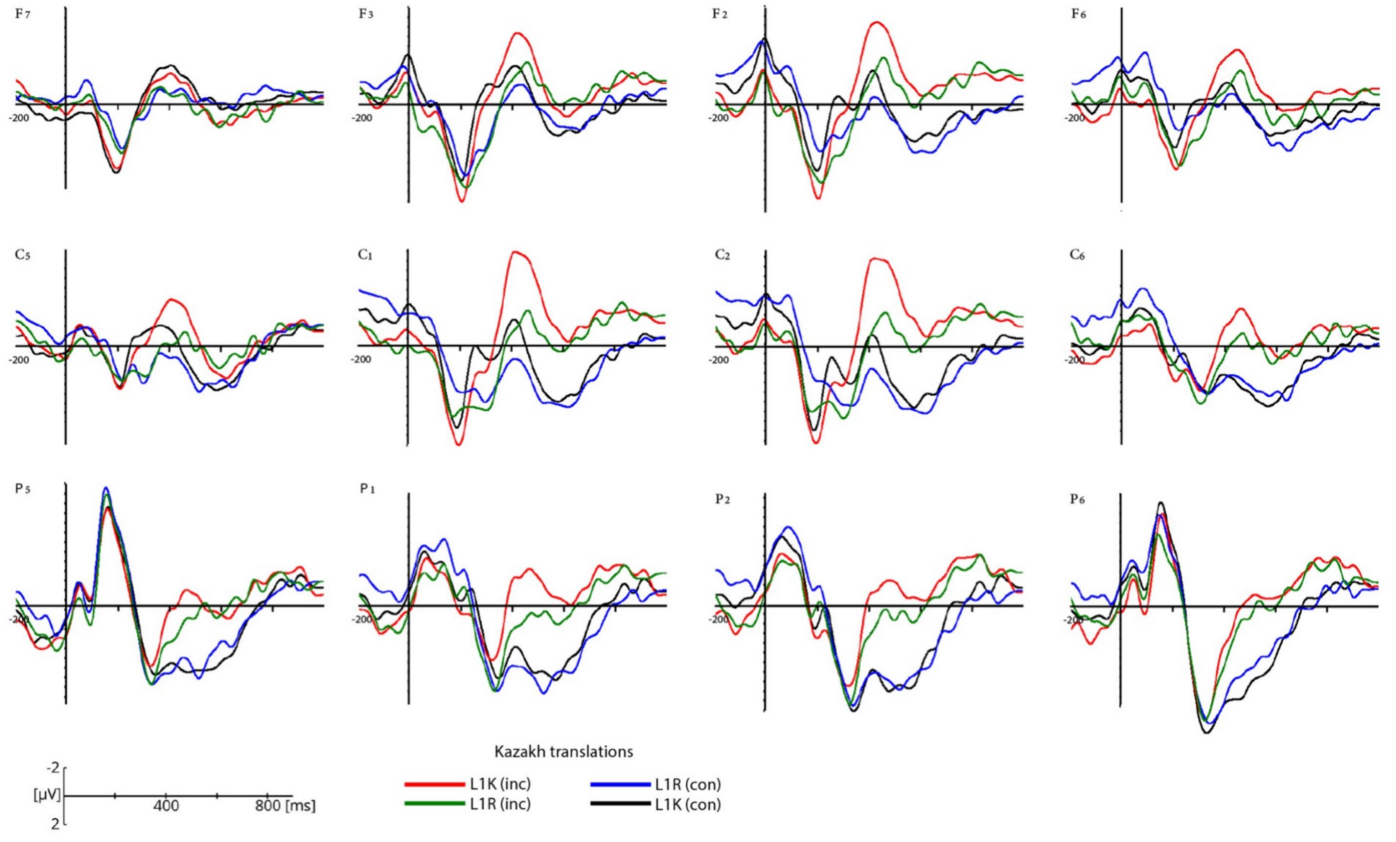
Grand average ERP waveforms for congruent Kazakh translations in the L1K group (black lines), congruent Kazakh translations in the L1R group (blue lines), and for incongruent Kazakh translations in the L1K group (red lines), for incongruent Kazakh translations in L1R group (green lines). ERPs are plotted for 12 electrodes (F7, F3, F2, F6, C5, C1, C2, C6, P5, P1, P2, P6) within each region of interest. The negative voltage is plotted up. Data are plotted from 200 ms prior to and 1,000 ms post stimulus onset.

**Figure 6:**
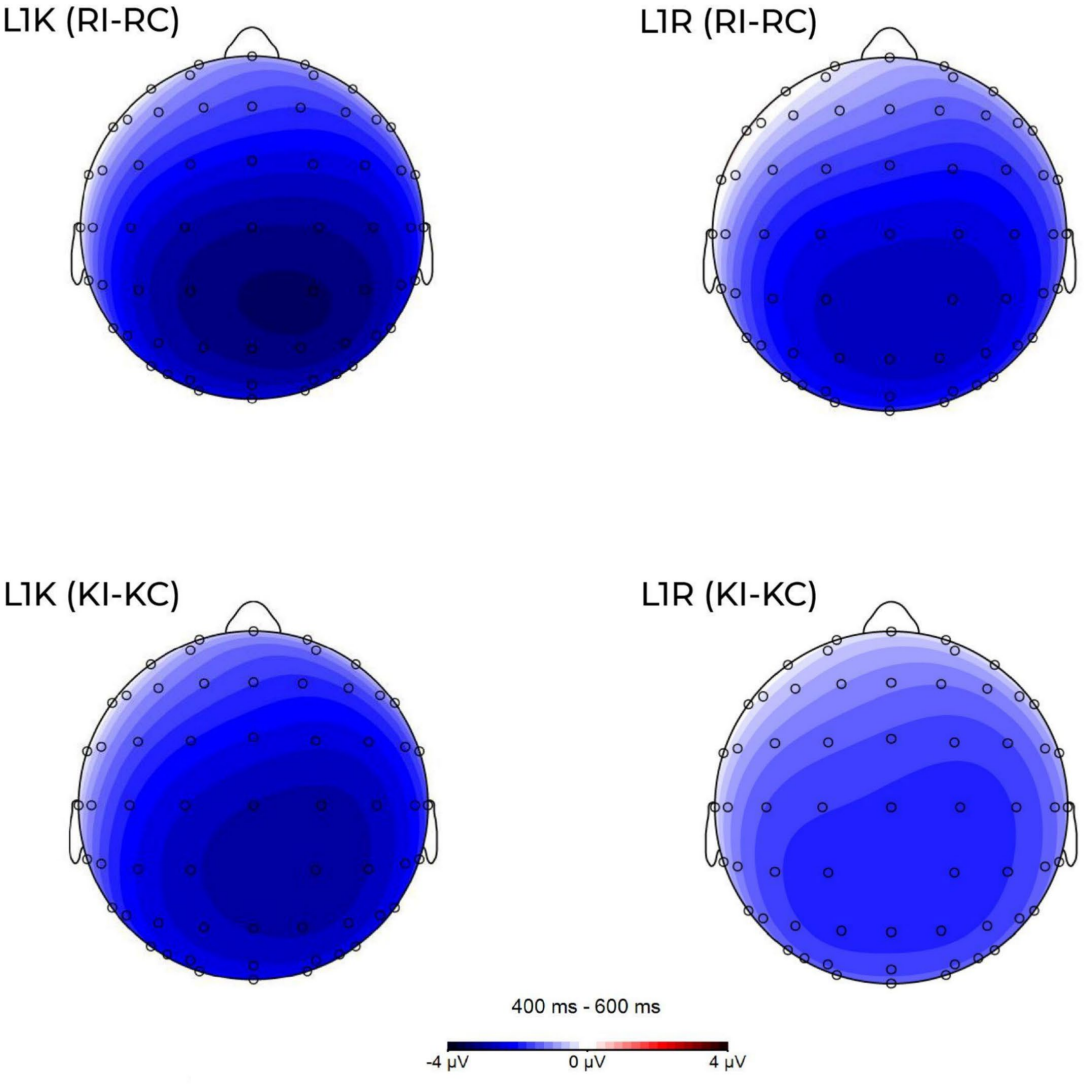
Scalp maps of condition effects (voltage differences were computed by subtracting the congruent from the incongruent conditions in Kazakh and Russian groups) averaged across the N400 (L1K and L1R (upper row), the incongruent condition and L1K and L1R (lower row), the translation equivalent condition). Scale from −4 μV (blue) to 4 μV (red).

Grand-average ERP waveforms in a selection of electrodes are shown for the congruent and incongruent Russian translations for L1R and L1K groups (Figure 4) and the congruent and incongruent Kazakh translations of within L1R and L1K (Figure 5). Figure 6 shows the scalp topographies of the mean amplitudes in the time windows for the N400 for the congruent translation condition (upper row) and the incongruent translation condition (lower row).

The participants from both groups showed a negative response to incongruent words in the 400-600 ms time window (see Figures 4–6). The difference in amplitude between the congruent and incongruent conditions was significant showing more prominent deflection on the incongruent conditions.

The ERP data were analyzed using linear mixed effects models as outlined below Table 1 provides a broad summary of effects in the N400 time window, respectively, using Type II Wald tests.

**Table 1:** Summary of effects in N400 time window (type II Wald tests).

Effect	Chisq	Df	Pr(>Chisq)
group	0.6068	1	0.436
cong	7060.8718	1	0.001
lang	9.0253	1	0.003
verb	409.7087	2	0.001
roi	19830.3820	8	0.001
group:cong	139.6412	1	0.001
group:lang	2.0607	1	0.151
cong:lang	4.2355	1	0.039
group:verb	629.4834	2	0.001
cong:verb	39.1927	2	0.001
lang:verb	52.6343	2	0.001
group:roi	1490.8787	8	0.001
cong:roi	790.5313	8	0.001
lang:roi	30.2510	8	0.001
verb:roi	251.5903	16	0.001
group:cong:lang	8.3207	1	0.003
group:cong:verb	8.4257	2	0.015
group:lang:verb	9.5682	2	0.008
cong:lang:verb	18.0522	2	0.001
group:cong:roi	17.3266	8	0.027
group:lang:roi	32.3764	8	0.001
cong:lang:roi	11.2709	8	0.187
group:verb:roi	101.6852	16	0.001
cong:verb:roi	7.1414	16	0.970
lang:verb:roi	8.7617	16	0.923
group:cong:lang:verb	0.5718	2	0.751
group:cong:lang:roi	3.2040	8	0.921
group:cong:verb:roi	0.4625	16	1.000
group:lang:verb:roi	0.5916	16	1.000
cong:lang:verb:roi	6.2649	16	0.985
group:cong:lang:verb:roi	0.8417	16	1.000

**Figure 7:**
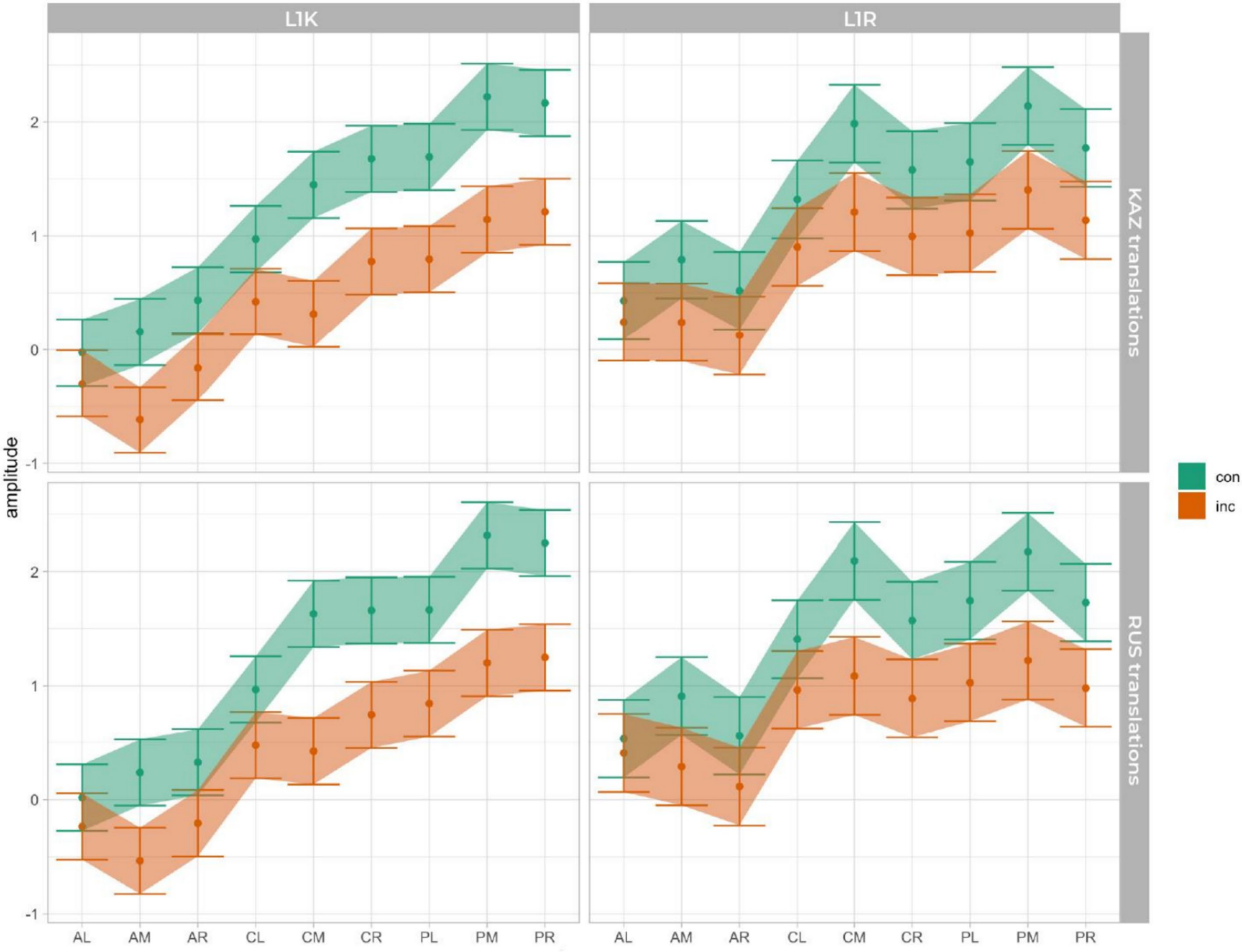
Estimated marginal means for the maximum amplitude in the N400 by group, language (language of translated words), ROI, and including the congruency.

In line with our hypotheses, we focus on interactions of group, condition and language of translations and for each statistical model, interpret the highest-order interaction involving both factors. This interaction is resolved and visualized in Figure 7, which shows estimated marginal means and 83% confidence intervals.

## Discussion

According to our hypothesis, it was anticipated that the N400 component would be increased, indicating inhibited recognition in incongruent condition. We believed this condition provided the strongest test of the hypothesis that the L1 and L2 were co-activated during L3 English processing, as it would require co-activation of the presented targets in both translation directions in the absence of any overt orthographic overlap with the presented L3 English words and between L1 and L2. To our knowledge, this study represents the first investigation into the translation recognition paradigm involving three languages (Kazakh, Russian, and English), with accompanying brain activity measurements, aimed at providing clarification of language distance in sequence of learning.

Regarding accuracy when judging in both conditions in L1s, we did not get a clear significant effect between groups or languages, whereas there is an interaction effect of congruency and group in L2 of the L1R group. Hence, it indicates an increased processing difficulty that led to more incorrect judgments in their L2 (Kazakh). However, there is little effect on reaction time without the main effect of group, which posits more or less equal time devotion to the processing in both groups (Figures 2, 3). In addition to participant-level factors, the behavioral models included stimulus-related predictors, and effects involving verb type were observed. Because verb type was not the primary theoretical focus of the present study and because all items were presented in the infinitive form to ensure cross-language comparability, we interpret these verb-related effects cautiously. The results nevertheless suggest that formal inflectional complexity may contribute to variability in translation recognition performance, and future studies that directly manipulate inflected verb forms and control lexical characteristics more tightly will be necessary to clarify these mechanisms.

The mean amplitude analysis between 400 and 600 ms revealed the significant effects in the similar ROIs between congruent and incongruent translations related to L1s of both groups: central middle (CM) and posterior middle (PM), which might indicate to the similar processing of L1s. Further, an interesting difference was obtained in L2s of both groups, where the same results were revealed in the ROIs for L2 of the L1K group, but the overlap observed in the L2 of the L1R group. The similarities in the ROIs might indicate the special status of L1 and differences could serve as evidence for the distance in language learning sequence (Figure 7). With the results obtained in this study, it is difficult to assume that translating from L3 to L2 or either from L3 to L1 could be based only on word association or words from the non-dominant languages cannot activate the corresponding conceptual node and provide a processing advantage to their L1 translations (see Kroll et al., 2002; Talamas et al., 1999; Stewart, 1994). For the L1 Russian group, the GLMM analysis revealed significant differences in N400 amplitude between congruent and incongruent translation equivalents in their first language (Russian) but not in their second language (Kazakh). This suggests that, unlike the L1 Kazakh group, the L1 Russian group exhibits weaker sensitivity to semantic congruency when processing translation equivalents in their L2. In other words, their neural response to lexical-semantic differences is pronounced only in their dominant language, while their L2 processing appears less affected by congruency. The restriction of the N400 effect to L1 highlights the asymmetric nature of bilingual lexical access in this group. This pattern may reflect differences in language dominance, proficiency, or exposure, suggesting that L2 Kazakh plays a less central role in their cognitive processing compared to their native Russian. The L1 Kazakh group’s stronger performance in both languages suggests greater bilingual proficiency, potentially stemming from differences in daily language usage and acquisition contexts.

The degree to which languages influence L3 processing can vary depending on the sequence in which they are learned. According to some research, learners may favorably depend on their L1 or L2 when it comes to cross-linguistic implications on L3 learning. Nevertheless, there is an ongoing debate about how these influences manifest and whether one of the background languages assumes the role of the ‘default supplier’ for cross-language influence (L1/L2) in L3 usage. Some cases suggest a strong preference toward one of the previously acquired languages as the primary source of cross-language influence in L3, such as studies indicating L1’s role in syntax and lexicon acquisition (e.g., Gollan et al., 2002; Angelovska and Hahn, 2012). Lindqvist (2009) discovered that, among three groups with different background language combinations, L1 predominantly influenced lexical aspects in L3 French acquisition.

Conversely, several L3 acquisition studies have highlighted cross-language influences stemming from the learner’s L2 (e.g., Ringbom, 1987; Hammarberg, 2001; Bardel and Falk, 2007; Fallah et al., 2016). The ‘L2 Status Factor” theory proposes that learners often activate the L2, rather than the L1, in L3 acquisition because the L2 shares more similarities with L3 concerning the learning context, age of onset, and metalinguistic knowledge (Bardel and Falk, 2007; Falk and Bardel, 2011). Furthermore, based on neurolinguistic claims (Ullman, 2005), Bardel and Falk (2012) suggested that both L2 and L3, as non-native languages, are stored in declarative memory, while the native language is stored in procedural memory.

Finally, new models raise doubts about how the sequence of acquisition affects L1 or L2’s privilege in cross-linguistic impacts on L3. The Scalpel Model (Slabakova, 2017) challenges the notion that languages previously learned have a universal impact on language acquisition during the early phases of learning. Instead, it suggests that cross-language influences may originate from either the L1 or the L2, or from both. Language learning is cumulative, and learners have access to all previously learned languages during L3 acquisition, according to the Linguistic Proximity Model (Westergaard et al., 2017; Westergaard, 2021).

The suggestion made subsequently by Potter et al. (1984) clarifies the link between the lexicons and whether or not they interact. Comparatively, the subordinate model suggests a bilingual structure in which the L2 (the subordinate language) and the L1 are directly connected at the lexical level, and the meaning of L2 words is nearly identical to that of L1 words. According to Weinreich (1953, p. 10), learning a new language using the ‘indirect method’ – that is, learning a language with the assistance of another is partially responsible for this structure, which together with the compound system, corresponds to the word association and concept mediation models, as well as Paradis’ (1980) proposed bilingual model, respectively.

Results of the L1R group clearly indicate the lack of an ‘indirect method’ in learning L3 (L1 → L2/L1 → L3), which could lead to dual activation of translations. Meanwhile, the group L1K indicates evidence of an ‘indirect method’ in learning L3 (L1 → L2 → L3), which retrieved translations equally according to the Revised Hierarchical Model (RHM) and facilitated the dual activation of translations. Further, it might mean that L3 of the L1K group is integrated with a single network containing lexical information and stored in a common lexicon with other languages.

It appears that L2 words in the L1R group receive less activation when an L3 word is encountered and vice versa because of weaker links between these two non-dominant languages, suggesting mediation by the native L1, if the model is correct and sublexical levels activate each time a Russian word is encountered (Gollan et al., 2005). This indicates that there is no facilitation produced by L3 words for L2, which may be due to their inability to transfer activation to the non-dominant language with a vast language distance in sequence of learning, according to our findings. The absence of code-switching effects seen in L3-L2 directions may also be explained by these. The findings of the research are slightly consistent in the group where L1 is Kazakh. These results demonstrate that there is parallel cross-language activation of semantic representations and, thus, that highly proficient bilinguals with Kazakh as L1 can have direct access to word meaning from the two languages in respect with the third language. Furthermore, the study highlighted that trilinguals who learned L3 through L2 (Kazakh) exhibited more pronounced effects in L2 relative to L1 (Russian), indicating the influence of language learning history on the activation of translation equivalents. The findings suggest that trilinguals learning L3 through L2 may develop stronger neural and cognitive associations with L2. However, further studies incorporating detailed language of instruction data are required to confirm this hypothesis.

The inquiry into whether bilingual individuals engage in selective or parallel activation of their two languages has garnered significant attention within the bilingual research community. Recent studies have increasingly provided support for the parallel access hypothesis (Grainger and Beauvillain, 1987; Beauvillain, 1992; Grainger and Dijkstra, 1992; Grainger, 1993; Li, 1996; Spivey and Marian, 1999; Marian and Spivey, 2003), substantiating the notion that bilinguals access their languages simultaneously rather than selectively. Our analysis of L1/L2 translations concerning L3 contributes to understanding cross-language interactions during multilingual processing. These findings align with models emphasizing the role of prior linguistic knowledge (e.g., Revised Hierarchical Model; Kroll and Stewart, 1994) and provide insights into the activation of previously acquired languages during L3 processing (cf. Westergaard et al., 2017; Bardel and Falk, 2012). While participants were matched based on their educational background and English proficiency, the absence of direct proficiency assessments, detailed language history data, and measures of cognitive factors such as working memory, limits the ability to attribute observed group differences solely to acquisition order or language distance. In addition, the language used during English (L3) instruction was not systematically recorded or controlled, which may have introduced additional variability in participants’ L3 learning histories and subsequent processing patterns. Future studies will incorporate standardized language assessments and comprehensive language background questionnaires to strengthen the validity of these comparisons.

## Conclusion

In conclusion, the results of the present study suggest that the imbalance between congruent translations and incongruent words in the translation recognition paradigm is affected by the language distance between L3 and L1/L2, which was affected by the sequence of language learning. When the distance is large and L2 is opaquer than L1, such as in the case of Russian-Kazakh-English trilinguals, a clear differentiation is not possible while processing the L2 Kazakh translation equivalents and incongruent words from L3, creating a linguistic *‘roundabout’* for L2 through L1 while processing L3. When the distance is small and L2 is similarly transparent as L1, creating a *‘forked route’* leading to the same lexico-semantic storage, such as in the case of Kazakh-Russian-English trilinguals, the existing N400 is sufficient in processing the translations from L3, implicating congruency. The present study provides important evidence for translation recognition by showing that the trilingual brain equally engages the same regions for different L1s.

An important strength of the present study lies in the language combination examined. Kazakh, Russian, and English differ substantially in typological structure, morphological complexity, and sociolinguistic status, providing a unique testing ground for models of multilingual language processing. Investigating this under-researched language constellation allows for the examination of cross-language activation patterns that may not be observable in more closely related language pairs, thereby extending existing models of bilingual and multilingual lexical processing.

Future research should explore the long-term effects of language learning sequence on cross-language activation and consider additional factors such as language proficiency and individual differences. Additionally, examining the implications of these findings for language education and cognitive training could provide valuable insights into optimizing multilingual learning strategies. A principal limitation of this research is the relatively small and imbalanced sample across groups, which constrains statistical power. Accordingly, our findings should be interpreted as preliminary and exploratory, rather than confirmatory. Nevertheless, the results highlight consistent N400 modulations that are theoretically meaningful and demonstrate the feasibility of investigating trilingual language processing in under-researched populations. We hope these findings will serve as a foundation for larger follow-up studies and for refining models of cross-linguistic influence in multilinguals.

## Data Availability

The datasets presented in this study can be found in online repositories. The names of the repository/repositories and accession number(s) can be found in the article/Supplementary material.

## References

[ref1] Alday P. M. (2018) lmerOut: LaTeX output for mixed effects models with lme4. Available online at: https://bitbucket.org/palday/lmerout/src/master/ (Accessed December 31, 2025).

[ref9001] Allen M. Poggiali D. Whitaker K. Marshall T. R. Kievit R. A. (2019). Raincloud plots: a multi-platform tool for robust data visualization. Wellcome Open Res. 4:63. doi: 10.12688/wellcomeopenres.15191.1 31069261 PMC6480976

[ref2] Alisharieva A. Ibraeva Z. Protasova E. (2017). Kazakhstani Russian: a view from the side. Ab Imperio 2017, 231–263. doi: 10.1353/imp.2017.0082, 34409987

[ref3] Angelovska T. Hahn A. (2012). “Written L3 (English): transfer phenomena of L2 (German) lexical and syntactic properties” in Cross-linguistic influences in multilingual language acquisition. ed. Gabrys-Barker D. (Berlin: Springer), 23–40.

[ref4] Aparicio X. Lavaur J. -M. (2014). Recognising words in three languages: effects of language dominance and language switching. Int. J. Multiling. 11, 164–181. doi: 10.1080/14790718.2013.783583

[ref5] Aparicio X. Lavaur J.-M. (2016). Masked translation priming effects in visual word recognition by trilinguals. J. Psycholinguist. Res. 45, 1369–1388. doi: 10.1007/s10936-015-9409-8, 26685863

[ref6] Balaguer R. D. D. Sebastián-Gallés N. Díaz B. Rodríguez-Fornells A. (2005). Morphological processing in early bilinguals: an ERP study of regular and irregular verb processing. Cogn. Brain Res. 25, 312–327. doi: 10.1016/j.cogbrainres.2005.06.003, 16023332

[ref7] Bardel C. Falk Y. (2007). The role of the second language in third language acquisition: the case of Germanic syntax. Second. Lang. Res. 23, 459–484. doi: 10.1177/0267658307080557

[ref8] Bardel C. Falk Y. (2012). “The L2 status factor and the declarative/procedural distinction” in Third language acquisition in adulthood. eds. Cabrelli Amaro J. Flynn S. Rothman J. (Amsterdam: John Benjamins), 61–78.

[ref9] Bates D. Mächler M. Bolker B. Walker S. (2015). Fitting linear mixed-effects models using lme4. J. Stat. Softw. 67, 1–48. doi: 10.18637/jss.v067.i01

[ref10] Beauvillain C. (1992). “Orthographic and lexical constraints in bilingual word recognition” in Cognitive processing in bilinguals. ed. Harris R. J. (Amsterdam: Elsevier), 221–235.

[ref11] Brysbaert M. New B. (2009). Moving beyond Kucera and Francis: a critical evaluation of current word frequency norms and the introduction of a New and improved word frequency measure for American English. Behav. Res. Methods 41, 977–990. doi: 10.3758/BRM.41.4.977, 19897807

[ref12] Bybee J. (1995). Regular morphology and the lexicon. Lang. Cogn. Process. 10, 425–455. doi: 10.1080/01690969508407111

[ref13] Chen H. C. Leung Y. S. (1989). Patterns of lexical processing in a nonnative language. J. Exp. Psychol. Learn. Mem. Cogn. 15, 316–325. doi: 10.1037/0278-7393.15.2.316

[ref14] De Groot A. M. B. (1992). Determinants of word translation. J. Exp. Psychol. Learn. Mem. Cogn. 18, 1001–1018. doi: 10.1037/0278-7393.18.5.1001

[ref15] Falk Y. Bardel C. (2011). Object pronouns in German L3 syntax: evidence for the L2 status factor. Second. Lang. Res. 27, 59–82. doi: 10.1177/0267658310386647

[ref16] Fallah N. Jabbari A. A. Fazilatfar A. M. (2016). Source (s) of syntactic cross linguistic influence (CLI): the case of L3 acquisition of English possessives by Mazandarani Persian bilinguals. Second. Lang. Res. 32, 225–245. doi: 10.1177/0267658315618009

[ref17] Fierman W. (2005). Kazakh language and prospects for its role in Kazakh “groupness”. Ab Imperio 2005, 393–423. doi: 10.1353/imp.2005.0065

[ref18] Fierman W. (2006). Language and education in post-soviet Kazakhstan: Kazakh-medium instruction in urban schools. Russ. Rev. 65, 98–116. doi: 10.1111/j.1467-9434.2005.00388.x

[ref19] Flynn S. Foley C. Vinnitskaya I. (2004). The cumulative enhancement model for language acquisition: comparing adults’ and children’s patterns of development in first, second and third language acquisition. Int. J. Multiling. 1, 3–17. doi: 10.1080/14790710408668175

[ref9002] Fox J. Weisberg S. (2011). An R Companion to Applied Regression, 2nd Edn. Thousand Oaks, CA: Sage.

[ref20] Fox J. Weisberg S. (2019). An R companion to applied regression. 3rd Edn. Thousand Oaks, CA, USA: Sage.

[ref21] Francis W. S. (2005). “Bilingual semantic and conceptual representation” in Handbook of bilingualism: Psycholinguistic approaches. eds. Kroll J. F. de Groot A. M. B. (Cambridge: Cambridge University Press), 251–267.

[ref22] Gollan T. H. Montoya R. I. Fennema-Notestine C. Morris S. K. (2005). Bilingualism affects picture naming but not picture classification. Mem. Cogn. 33, 1220–1234. doi: 10.3758/BF03193224, 16532855

[ref23] Gollan T. H. Montoya R. I. Werner G. A. (2002). Semantic and letter fluency in Spanish-English bilinguals. Neuropsychology 16, 562–576. doi: 10.1037/0894-4105.16.4.562, 12382994

[ref24] Grainger J. (1993). “Visual word recognition in bilinguals” in The bilingual lexicon. eds. Schreuder R. Weltens B. (Amsterdam: John Benjamins), 11–26.

[ref25] Grainger J. Beauvillain C. (1987). Language blocking and lexical access in bilinguals. Q. J. Exp. Psychol. 39, 295–320.

[ref26] Grainger J. Dijkstra A. (1992). “On the representation and use of language information in bilinguals” in Cognitive processing in bilinguals. ed. Harris R. J. (Amsterdam: Elsevier), 207–220.

[ref27] Hammarberg B. (2001). “Roles of L1 and L2 in L3 production and acquisition” in Cross-linguistic influence in third language acquisition: Psycholinguistic perspectives. eds. Cenoz J. Hufeisen B. Jessner U. (Bristol: Multilingual Matters), 21–41.

[ref29] Jaensch C. (2011). L3 acquisition of German adjectival inflection – a generative account. Second. Lang. Res. 27, 83–105. doi: 10.1177/0267658310386646

[ref30] Keatley C. Spinks J. De Gelder B. (1992). Asymmetrical semantic facilitation between languages: Evidence for separate representational systems in bilingual memory [unpublished manuscript]. Tilburg: University of Tilburg.

[ref31] Kroll J. F. (1993). “Accessing conceptual representation for words in a second language” in The bilingual lexicon. eds. Schreuder R. Weltens B. (Amsterdam: John Benjamins), 53–81.

[ref32] Kroll J. F. Curley J. (1988). “Lexical memory in novice bilinguals: the role of concepts in retrieving second language words” in Practical aspects of memory. eds. Gruneberg M. Morris P. Sykes R., vol. 2 (Hoboken, NJ: Wiley), 389–395.

[ref33] Kroll J. F. Michael E. Tokowicz N. Dufour R. (2002). The development of lexical fluency in a second language. Second. Lang. Res. 18, 137–171. doi: 10.1191/0267658302sr201oa

[ref34] Kroll J. F. Sholl A. (1992). “Lexical and conceptual memory in fluent and nonfluent bilinguals” in Cognitive processing in bilinguals. ed. Harris R. J. (Amsterdam: Elsevier), 191–204.

[ref35] Kroll J. F. Stewart E. (1990). Concept mediation in bilingual translation. Paper presented at the 31st annual meeting of the Psychonomic society. New Orleans.

[ref36] Kroll J. F. Stewart E. (1994). Category interference in translation and picture naming: evidence for asymmetric connection between bilingual memory representations. J. Mem. Lang. 33, 149–174. doi: 10.1006/jmla.1994.1008

[ref37] Kroll J. F. Tokowicz N. (2005) Models of bilingual representation and processing: looking back and to the future Kroll J. F. Groot A. M. B. De Handbook of bilingualism: Psycholinguistic approaches 531–553 Oxford: Oxford University Press

[ref38] Kroll J. F. Van Hell J. G. Tokowicz N. Green D. W. (2010). The revised hierarchical model: a critical review and assessment. Biling. Lang. Congn. 13, 373–381. doi: 10.1017/S136672891000009X, 20676387 PMC2910435

[ref39] Kutas M. Federmeier K. D. (2011). Thirty years and counting: finding meaning in the N400 component of the event-related brain potential (ERP). Annu. Rev. Psychol. 62, 621–647. doi: 10.1146/annurev.psych.093008.131123, 20809790 PMC4052444

[ref40] Laxén J. Lavaur J.-M. (2010). The role of semantics in translation recognition: effects of number of translations, dominance of translations, and semantic relatedness of multiple translations. Biling. Lang. Cogn. 13, 157–183. doi: 10.1017/S1366728909990472

[ref41] Lenth R. (2019) Emmeans: estimated marginal means, aka least-squares means. Available online at: https://CRAN.R-project.org/package=emmeans (Accessed December 31, 2025).

[ref42] Li P. (1996). Spoken word recognition of code-switched words by Chinese-English bilinguals. J. Mem. Lang. 35, 757–774. doi: 10.1006/jmla.1996.0039

[ref43] Lindqvist C. (2009). The use of the L1 and the L2 in French L3: examining cross-linguistic lexemes in multilingual learners’ oral production. Int. J. Multiling. 6, 281–297. doi: 10.1080/14790710902812022

[ref44] Marian V. Spivey M. (2003). Competing activation in bilingual language processing: within and between-language competition. Biling. Lang. Cogn. 6, 97–115. doi: 10.1017/S1366728903001068

[ref45] Paradis M. (1980). “Language and thought in bilinguals” in The sixth LACUS forum 1979. eds. McCormack W. C. Izzo H. J. (Essex: Horn Beam Press), 420–431.

[ref46] Paradis M. (1994). “Neurolinguistic aspects of implicit and explicit memory: implications for bilingualism” in Implicit and explicit learning of second languages. ed. Ellis N. (Cambridge, MA: Academic Press), 393–419.

[ref47] Paradis M. (2004). A neurolinguistic theory of bilingualism. Amsterdam: John Benjamins.

[ref48] Paradis M. (2009). Declarative and procedural determinants of second languages. Studies in bilingualism, vol. 40. Amsterdam: John Benjamins.

[ref49] Pinker S. Ullman M. T. (2002). The past and future of the past tense. Trends Cogn. Sci. 6, 456–463. doi: 10.1016/S1364-6613(02)01990-3 12457895

[ref50] Potter M. C. So K. F. Von Eckardt B. Feldman L. B. (1984). Lexical and conceptual representation in beginning and proficient bilinguals. J. Verbal Learn. Verbal Behav. 23, 23–38. doi: 10.1016/S0022-5371(84)90489-4

[ref51] Ringbom H. (1987). The role of the first language in foreign language learning. Multilingual Matters 5, 244–245. doi: 10.1177/026565908900500218, 41459393

[ref52] Roufca P. (1992). A longitudinal study of second language acquisition in French [unpublished manuscript]. South Hadley, MA: Mount Holyoke College.

[ref9004] RStudio Team (2021). RStudio: Integrated Development Environment for R. RStudio, PBC, Boston, MA, USA.

[ref53] Sánchez-Casas R. M. Davis C. W. Garcia-Albea J. E. (1992). Bilingual lexical processing: exploring the cognate-noncognate distinction. Eur. J. Cogn. Psychol. 4, 293–310. doi: 10.1080/09541449208406189

[ref54] Schad D. J. Vasishth S. Hohenstein S. Kliegl R. (2020). How to capitalize on a priori contrasts in linear (mixed) models: a tutorial. J. Mem. Lang. 110:104038. doi: 10.1016/j.jml.2019.104038

[ref55] Sholl A. Sankaranarayanan A. Kroll J. F. (1995). Transfer between picture naming and translation: a test of asymmetries in bilingual memory. Psychol. Sci. 6, 45–49. doi: 10.1111/j.1467-9280.1995.tb00303.x

[ref56] Slabakova R. (2017). The scalpel model of third language acquisition. Int. J. Bil. 21, 651–665. doi: 10.1177/1367006916655413

[ref57] Smagulova J. (2021). When language policy is not enough. Int. J. Sociol. Lang. 2021, 265–269. doi: 10.1515/ijsl-2020-0101

[ref58] Spivey M. Marian V. (1999). Cross talk between native and second languages: partial activation of an irrelevant lexicon. Psychol. Sci. 10, 281–284. doi: 10.1111/1467-9280.00151

[ref9005] Stewart A. L. (1994). The reliability and validity of self-reported health status measures. In: Stewart A. L. Ware J. E. (eds) Measuring Functioning and Well-Being: The Medical Outcomes Study Approach. Durham, NC: Duke University Press, 345–371.

[ref59] Suleimenova E. D. (2009). Language policy and dynamics of the language situation in Kazakhstan. Russ. Lang. J. 59, 21–36.

[ref60] Suleimenova E. D. (2011). Language processes and policy: Monograph. Almaty: Kazakh University.

[ref61] Suleimenova E. D. (2020). Russian overseas flagship’ and language situation in Kazakhstan. Russian Lang. J. 70, 23–58.

[ref62] Talamas A. Kroll J. F. Dufour R. (1999). Form related errors in second language learning: a preliminary stage in the acquisition of L2 vocabulary. Bilingual. Lang. Cogn. 2, 45–58.

[ref63] Ullman M. (2005). “A cognitive neuroscience perspective on second language acquisition: the declarative/procedural model” in Mind and context in adult second language acquisition: Methods, theory, and practice. ed. Sanz C. (Washington, DC: Georgetown University Press), 141–178.

[ref64] Weinreich U. (1953). Languages in contact. New York: The Linguistic Circle of New York.

[ref65] Westergaard M. (2021). Microvariation in multilingual situations: the importance of property-by-property acquisition. Second. Lang. Res. 37, 379–407. doi: 10.1177/0267658319884116

[ref66] Westergaard M. Mitrofanova N. Mykhaylyk R. Rodina Y. (2017). Crosslinguistic influence in the acquisition of a third language: the linguistic proximity model. Int. J. Bilingual. 21, 666–682. doi: 10.1177/1367006916648859

[ref67] Wickham H. Averick M. Bryan J. Chang W. McGowan L. D.v A. François R. . (2019). Welcome to the Tidyverse. J. Open Source Softw. 4:1686. doi: 10.21105/joss.01686

[ref68] Wilke C. O. (2019) Cowplot: Streamlined plot theme and plot annotations for ‘ggplot2’. Available online at: https://CRAN.R-project.org/package=cowplot (Accessed December 31, 2025).

[ref69] Zhu H. (2019) KableExtra: Construct complex Table with ‘kable’ and pipe syntax. Available online at: https://CRAN.R-project.org/package=kableExtra (Accessed December 31, 2025).

