# Supplementary material for: Processing effects of L1/L2 from L3 in translation recognition paradigm: an exploratory ERP study
Source: Front Psychol. 2026 Jan 13;16:1710959. doi: 10.3389/fpsyg.2025.1710959 (PMC12835209; doi:10.3389/fpsyg.2025.1710959)
Supplement: Supplementary file 1 [file Table_1.docx]

# Appendix 1

| **№** | **English verbs** | **Kazakh Congruent Translation** | **Kazakh Incongruent Translation** | **Russian Congruent Translation** | **Russian Incongruent Translation** |
| --- | --- | --- | --- | --- | --- |
| **1.** | come | келу | көшу | приходить | пугать |
| **2.** | find | табу | тасу | находить | нарушать |
| **3.** | drink | ішу | ілу | пить | петь |
| **4.** | let | рұқсат беру | ретсіз кіру | разрешать | расстилать |
| **5.** | hit | ұру | ұту | ударить | удалять |
| **6.** | broadcast | баяндау | бағдарлау | вещать | вешать |
| **7.** | show | көрсету | көтеру | показывать | покалывать |
| **8.** | wet | ылғалдандыру | ыңғайсыздану | увлажнять | уважать |
| **9.** | smell | иіскеу | иіру | нюхать | нудить |
| **10.** | leave | қалдыру | қолдану | оставить | отдавать |
| **11.** | learn | оқу | ою | учить | усечь |
| **12.** | catch | ұстап алу | ұштастыру | ловить | ладить |
| **13.** | burst | жарылу | жабылу | лопнуть | лопать |
| **14.** | know | білу | бүру | знать | зреть |
| **15.** | say | айту | аңду | сказать | скакать |
| **16.** | ring | шырылдау | шырыштау | звонить | зубрить |
| **17.** | take | алу | ашу | взять | врать |
| **18.** | mow | ору | орау | косить | копить |
| **19.** | knit | тоқу | толу | вязать | вянуть |
| **20.** | quit | тастау | тосу | бросать | бродить |
| **21.** | wear | кию | күлу | носить | нежить |
| **22.** | hear | есту | есу | слушать | служить |
| **23.** | grow | өсу | өлу | расти | рассечь |
| **24.** | slide | сырғанау | сыбырлау | скользить | скрепить |
| **25.** | sell | сату | сақтау | продавать | помогать |
| **26.** | swell | ісіну | іздену | распухать | раскрывать |
| **27.** | sing | өлең айту | өкпе айту | петь | пасть |
| **28.** | tear | жырту | жығу | разрывать | разбивать |
| **29.** | shear | қырқу | қысу | стричь | счесть |
| **30.** | lose | жоғалту | жорғалау | терять | терпеть |
| **31.** | slit | кесу | кету | разрезать | разделить |
| **32.** | recast | түрлендіру | түрегелу | видоизменять | владычествовать |
| **33.** | feed | тамақтандыру | тұрақтандыру | кормить | корчить |
| **34.** | creep | еңбектеу | егелеу | ползти | потеть |
| **35.** | steal | ұрлау | ұштау | красть | крыть |
| **36.** | underbid | бағаны төмендету | баяндама тапсыру | сбивать цену | сброшюровать |
| **37.** | seek | іздеу | ілу | искать | испечь |
| **38.** | saw | аралау | арлану | пилить | плакать |
| **39.** | get | алу | азу | получить | приучать |
| **40.** | sew | тігу | тізу | шить | жить |
| **41.** | sink | бату | бағу | тонуть | терпеть |
| **42.** | run | жүгіру | жеткізу | бежать | белить |
| **43.** | go | жүру | жету | идти | играть |
| **44.** | spend | өткізу | өтіну | тратить | травить |
| **45.** | lie | жату | жамау | лежать | легчать |
| **46.** | cast | лақтыру | лапылдау | кидать | кивать |
| **47.** | dream | армандау | арқалау | мечтать | молчать |
| **48.** | shut | жабу | жату | закрывать | задавать |
| **49.** | begin | бастау | баптау | начать | нажать |
| **50.** | inset | қондыру | қостыру | вставлять | вставать |
| **51.** | break | сындыру | секіру | сломать | создать |
| **52.** | sleep | ұйықтау | ұмыту | спать | спечь |
| **53.** | win | жеңу | жегу | выиграть | выявлять |
| **54.** | throw | тастау | таптау | бросить | бродить |
| **55.** | upset | ренжіту | рухтану | огорчать | ограждать |
| **56.** | outbid | қайта сатып алу | қарсылық таныту | перекупать | перечитать |
| **57.** | sweep | сыпыру | сұрыптау | подметать | потребить |
| **58.** | fall | құлау | құрау | падать | палить |
| **59.** | spread | тарату | түсіну | распространять | регистрировать |
| **60.** | have | ие болу | икемдену | иметь | издать |
| **61.** | burn | жағу | жұту | жечь | жать |
| **62.** | build | құрастыру | құшақтасу | строить | спилить |
| **63.** | cost | тұру баға | тығу | стоить | строчить |
| **64.** | see | көру | көктеу | видеть | витать |
| **65.** | stand | тұру | түлеу | стоять | стрелять |
| **66.** | wed | үйлену | үйрену | жениться | жертвовать |
| **67.** | cut | кесу | керу | резать | решать |
| **68.** | bite | тістеу | тыстау | кусать | кутить |
| **69.** | bend | бүгілу | бөлісу | согнуть | собрать |
| **70.** | reset | қайта орнату | құпия сақтау | вправлять | вписать |
| **71.** | split | жару | жаттау | колоть | корать |
| **72.** | spoil | бүлдіру | бекіну | портить | познать |
| **73.** | hew | шабу | шағу | рубить | решать |
| **74.** | forecast | болжамдау | бекемдеу | прогнозировать | пронумеровать |
| **75.** | overcast | бүркену | бүлдіру | покрыться | погреться |
| **76.** | make | жасау | жару | делать | делить |
| **77.** | keep | сақтау | санау | хранить | храпеть |
| **78.** | sweat | терлеу | торлау | потеть | потечь |
| **79.** | shed | төгу | тебу | пролить | пробить |
| **80.** | rid | құтылу | құрғату | избавляться | избаловать |
| **81.** | feel | сезу | сыру | чувствовать | чествовать |
| **82.** | thrust | итеру | игеру | толкать | топтать |
| **83.** | swim | жүзу | жұту | плавать | плавить |
| **84.** | spit | түкіру | түсіру | плевать | пачкать |
| **85.** | think | ойлау | оқтау | думать | дубить |
| **86.** | bust | бұзу | безу | взломать | взвесить |
| **87.** | dig | қазу | қосу | копать | коптеть |
| **88.** | sow | себу | септеу | сеять | сереть |
| **89.** | fit | сәйкес келу | сықылықтау | ссоответствовать | совершенствовать |
| **90.** | blow | үрлеу | үсу | дуть | драть |
| **91.** | hurt | жаралау | жалығу | ранить | родить |
| **92.** | strew | шашу | шалу | сыпать | сыскать |
| **93.** | spring | секіру | сепкілеу | прыгнуть | прыснуть |
| **94.** | bet | бәстесу | бекіту | держать пари | деревенеть |
| **95.** | hold | ұстау | ұрлау | держать | дергать |
| **96.** | telecast | тарату | тоқтату | передавать | переделать |
| **97.** | forget | ұмыту | ұмтылу | забыть | зевать |
| **98.** | choose | таңдау | тоқтау | выбрать | воевать |
| **99.** | set | орнату | отыру | устанавливать | усиливаться |
| **100.** | earn | табыс табу | таныс табу | зарабатывать | зарумянивать |
| **101.** | fight | күресу | керілу | драться | дуться |
| **102.** | offset | ығыстыру | ынталану | смещать | сменить |
| **103.** | beset | қоршау | қорғау | окружать | окрылять |
| **104.** | hide | тығылу | таңырқау | прятать | прыснуть |
| **105.** | buy | сатып алу | сарғылттану | купить | кутать |
